# A Bootstrap Framework for Aggregating within and between Feature Selection Methods

**DOI:** 10.3390/e23020200

**Published:** 2021-02-06

**Authors:** Reem Salman, Ayman Alzaatreh, Hana Sulieman, Shaimaa Faisal

**Affiliations:** Department of Mathematics and Statistics, American University of Sharjah, Sharjah P.O. Box 26666, United Arab Emirates; g0007108@aus.edu (R.S.); hsulieman@aus.edu (H.S.); g00085281@aus.edu (S.F.)

**Keywords:** ensemble learning, feature selection, mean aggregation, entropy, stability

## Abstract

In the past decade, big data has become increasingly prevalent in a large number of applications. As a result, datasets suffering from noise and redundancy issues have necessitated the use of feature selection across multiple domains. However, a common concern in feature selection is that different approaches can give very different results when applied to similar datasets. Aggregating the results of different selection methods helps to resolve this concern and control the diversity of selected feature subsets. In this work, we implemented a general framework for the ensemble of multiple feature selection methods. Based on diversified datasets generated from the original set of observations, we aggregated the importance scores generated by multiple feature selection techniques using two methods: the Within Aggregation Method (WAM), which refers to aggregating importance scores within a single feature selection; and the Between Aggregation Method (BAM), which refers to aggregating importance scores between multiple feature selection methods. We applied the proposed framework on 13 real datasets with diverse performances and characteristics. The experimental evaluation showed that WAM provides an effective tool for determining the best feature selection method for a given dataset. WAM has also shown greater stability than BAM in terms of identifying important features. The computational demands of the two methods appeared to be comparable. The results of this work suggest that by applying both WAM and BAM, practitioners can gain a deeper understanding of the feature selection process.

## 1. Introduction

Over the years, feature selection has become a fundamental preprocessing tool in data mining and machine learning. Otherwise known as attribute or variable selection, feature selection refers to the process of reducing the number of input variables for predictive modeling. The objective of feature selection is three-fold: improving the prediction performance of the features, providing faster and more cost-effective features and gaining a better understanding of the underlying process that generated the data. This objective is achieved through the characterization and elimination of irrelevant and redundant features, leaving only a subset of the most useful features to be used in further analysis. As such, features represent the individual independent variables typically used as predictors within a model. Throughout this paper, we use the terms variable and feature exchangeably.

There are generally three classes of feature selection methods based on how the method interacts with the learning algorithm: filter, wrapper and embedded methods [1]. Filter methods evaluate the importance of features as a pre-processing operation to the learning algorithm and select the best feature subsets through some information metrics without direct input from the target variable. Filters are known to have high computational efficiency compared to the wrapper and embedded methods. Alternatively, the wrapper methods apply some search in the feature space and use the learning algorithm to evaluate the importance of feature subsets. Thus, wrappers are deemed to be computationally expensive and can be slow due to the need to apply the learning algorithm to each new feature subset. The embedded methods perform the feature selection internally to the learning algorithm. In this approach, a predefined importance criterion is integrated into the learning algorithm, and features which meet the set criteria are selected. Embedded methods have less computational cost than wrappers but are more likely to suffer from over-fitting.

One of the most common challenges encountered in feature selection is choosing the most suitable method for a given problem. In general, there is no single feature selection method that outperforms all others across most applications. The three different classes of feature selection methods are frequently suitable under varying conditions. For instance, the greedy randomized adaptive heuristic (GRASP) filter technique introduced in [2] provides efficient results for problems with a large number of nominal features or with simulated instances, when the bias introduced by the classification methods is minimized. In this context, an overall understanding of various types of feature selection algorithms is often needed before an appropriate method selection could be made. Often, such a choice is based on massive empirical evaluations of diverse feature selection methods ([3,4]). Alternatively, meta-learning and ensemble methods are two widely used approaches for determining the most appropriate feature selection algorithm for a problem or circumventing it entirely.

Meta-learning predicts the most appropriate feature selection method for a given dataset. In meta-learning, the feature selection task is treated as a supervised learning problem in which the datasets are objects and the target function maps a dataset to the feature selection method that shows the highest performance based on a certain criterion. With the increase in feature selection techniques and introduction of new methods, the implementation of meta-learning can provide a valuable way of weighing the most suitable feature selection methods. However, like other learning techniques, in order to make reliable recommendations, metalearning can be limited by its need for a suitable metadatabase that is representative of the given problem domain [5]. An excellent review of meta-learning algorithms can be found in [6].

Ensemble methods were originally developed to enhance classification performance [7]. Ensemble feature selection has two main components: diversification, to create varying feature selection outputs; and aggregation, to combine the generated outputs. Diversification can be achieved by data resampling [8]. In data resampling, several randomly generated subsamples are drawn from the original dataset, and then a feature selection technique is applied on each generated subsample. The ensemble method combines the results generated by each feature selection method [9]. Bootstrap sampling is commonly used to generate the random subsamples [10,11,12]. When the amount of available data is sufficient, in [13], the partitioning of the data into non-overlapping chunks was proposed.

Although a number of aggregation techniques have been proposed in the literature, there is no clear rule to determine which one of them should be chosen for a specific feature selection task. However, the simple approach of mean-based aggregation seem to be efficient and compelling in most cases [14]. In [15], Kolde et al. (2012) proposed a novel rank aggregation method based on order statistics and applied it to gene selection. The approach detects genes (features) that are ranked consistently better than the expected behavior of uncorrelated features and assigns a significance score to each gene. In [16], Ditzler et al. (2014) developed a statistical testing framework in which a statistical test is performed for the number of times that each feature has been selected to determine whether it belongs to the relevant feature set or not.

The robustness or stability of the feature selection method is of paramount importance to reducing dimensionality and improving the performance of the learning algorithm. Stability measures the insensitivity of the feature selection method to variations in the training set. In other words, unstable feature selection methods can produce varied feature rankings when a single feature selection technique is applied to different training samples generated from the same dataset. Instability can also occur when very different feature selection techniques applied to the same dataset produce different feature rankings. The stability of feature selection has attracted a plethora of research in machine learning and data mining communities over the last decade [8]. It has become crucial to supplement the investigation of model performance with stability analysis in order to ensure the quality of feature selection [17].

This paper introduces a general framework for a bootstrap ensemble in which feature selection results are aggregated within and between multiple feature selection methods. Multiple subsamples are generated from the original dataset by bootstrapping, and the feature selection techniques are applied on each subsample. The aggregation is thus two-fold; first, the single feature selection method is aggregated across subsamples, and second, different feature selection methods are combined into a single set. The mean-based aggregation rule is used where the generated importance scores are combined within and between the feature selection methods. Because filter feature selection methods are known for their computational efficiency, we chose four traditional filter techniques for our experimental framework, although wrappers and embedded methods are also applicable. Our experimental results with real data sets demonstrated that ensembling multiple feature selection methods improves the performance of the learning algorithm, guides the selection of the optimal feature subset and facilitates the identification of the most appropriate feature selection method for a given dataset. Furthermore, comparisons of the two folds of aggregation revealed that, on the whole, aggregating within a single feature selection method outperforms aggregating between multiple feature selection methods.

The rest of this paper is organized as follows. In Section 2, we introduce the general framework for the bootstrap aggregation within and between multiple feature selection methods. In Section 3, we describe the robustness metrics used for the evaluation of the feature selection stability. In Section 4, we present and analyze the results of our experimental work. Finally, conclusions and some insights into future work are presented in Section 5.

## 2. Bootstrap Aggregation Framework

Let us consider a dataset S≡(X,Y), with *n* observations and *p* features such that $n,p\in Z_{>0}$; that is, $X={\left[x_{ij}\right]}_{n\times p}\in R^{n,p}$ is the matrix of observations and *Y* is the target variable (i.e., the rows are the observations and the columns are the variables). Moreover, $x_{ij}$ denotes observation *i* of the feature *j*. The goal of this work is to reduce the number of features in the dataset X in order to predict the target variable Y. Now, let $\left\{V_{1},\ldots ,V_{p}\right\}$ denote the set of features (variables) in X. The dataset S is divided into a training dataset X, and a testing dataset T. Here, $X=X_{\lceil rn\rceil ,p}$ and $T=X_{n-\lceil rn\rceil ,p}$ with 0<r<1. For instance, considering the Philippine dataset from Table 1, Philippine has n = 5832 observations and p = 309 features. Moreover, $\left\{V_{1},\ldots ,V_{309}\right\}$ denote the set of features in the Philippine dataset. Two-thirds of the Philippine dataset is taken for training whereas one-third is used for testing. Then, the training data are denoted as $X=X_{3888,309}$ and the testing data are denoted as $T=X_{1944,309}$. We use feature selection to reduce the 309 features in the Philippine dataset, leading to the dataset only containing the most relevant features.

Let $FS_{1},\cdots ,FS_{t}$ denote the feature selection methods used, where $t\in Z_{>0}$. In addition, we assume that each feature selection method $FS_{q}\in \left\{FS_{1},\ldots ,FS_{t}\right\}$ generates a feature importance score $\ell_{j}\in R$ for every feature $V_{j}\in \left\{V_{1},\ldots ,V_{p}\right\}$. Although rank aggregation could also be used for the feature selection process as an alternative to score aggregation, aggregating the ranks from different feature selection algorithms might result in ties. For instance, three feature selection algorithms might rank one feature as 2, 1 and 3 and another feature as 2, 3 and 1. The average rank for both features would be 2. A merit of this study is that it uses the importance scores to aggregate feature selection methods, as the scores have a stronger scale than the rank and can better differentiate between the features. Furthermore, averaging the actual importance scores is simple to implement and perform. For the meaningful comparison of the scores derived from different feature selection algorithms, a normalization technique has been implemented. In this section, we discuss the use of bootstrap techniques in order to combine the feature importance scores within each $FS_{q}$ and between $FS_{q}$ for all q∈{1,…,t}. For the convenience of presentation and interpretation, the following terminologies are used in the rest of the paper:

- The Within Aggregation Method (WAM) refers to aggregating importance scores within a single feature selection method.
- The Between Aggregation Method (BAM) refers to aggregating importance scores between different feature selection methods.

Due to the simplicity and the efficiency of the arithmetic mean [14], it is used in this paper to aggregate the feature importance scores resulting from the different feature selection methods. However, the proposed framework allows the application of other score or rank aggregation rules, including the median, geometric mean and others.

### 2.1. Feature Selection Based on WAM

**Figure 1:**
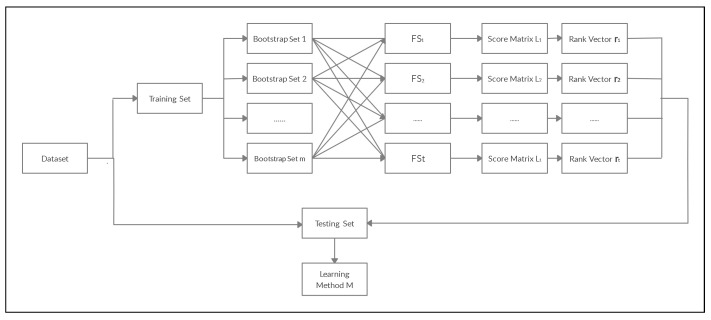
Framework for the Within Aggregation Method (WAM).

Let us consider a training dataset X and a feature selection method FS. Let $X_{1},$$X_{2},$$\ldots ,X_{m}$ be bootstrapped samples from X, where $m\in Z_{>0}.$ Then, we apply FS on each $X_{s}$, s=1,⋯,m, which in turn generates feature scores $\left\{\ell_{s1},\ldots ,\ell_{sp}\right\}$ that correspond to the set of features $\{V_{1},\ldots ,V_{p}\}.$ Therefore, a score matrix $L=\left[\ell_{sj}\right]\in R^{m\times p}$ is generated after applying the feature selection method FS on each bootstrap sample. In L, column *j* represents the FS importance scores for variable $V_{j}$ over the *m* bootstrap sample datasets. The final aggregated score for the feature $V_{j}$ is defined to be the mean of column *j* in L. We use the notation $\bar{\ell_{.j}}=\frac{\sum_{s=1}^{m}\ell_{sj}}{m}$ to denote the aggregated scores of $V_{j}.$ Then, a rank vector $r=\left(r_{1},\ldots ,r_{p}\right),\;r_{j}\in \left\{1,2,\ldots ,p\right\}$ is assigned to the feature set $\left\{V_{1},\ldots ,V_{p}\right\}$ based on the aggregated scores $\{\bar{\ell_{.1}},\ldots ,\bar{\ell_{.p}\}}.$ The feature set is then sorted from the most to the least important based on the rank vector r. Now, based on a threshold parameter, 0<k≤1, we keep only the most important 100k% of the feature set (determined by the rank vector r). The WAM approach can be used to compare the performance of different feature selection techniques based on various supervised learning methods for a given dataset. The flowchart in Figure 1 explains the WAM. The following gives the associated computational Algorithm 1.
**Algorithm 1WAM Algorithm:**Given a training dataset X with *p* features, a testing dataset T, a feature selection method FS, a threshold parameter k, and a learning algorithm M.
(i)For s=1,…,m, generate bootstrap samples, $X_{1},\ldots ,X_{m}$ of the training dataset X.(ii)Based on FS, get the features score matrix L.(iii)Get the aggregated score set $\{{\bar{\ell }}_{.1},\ldots ,{\bar{\ell }}_{.p}\}.$(iv)For the aggregated score set $\{{\bar{\ell }}_{.1},\ldots ,{\bar{\ell }}_{.p}\},$ get the corresponding rank vector $r=(r_{1},\ldots ,r_{p}).$(v)Based on the rank vector r, keep only the top 100k% of the variable set $\{V_{1},\ldots ,V_{p}\}.$(vi)Based on the selected feature set in (v), use the testing dataset T and a cross-validation technique to train and test the model M.

### 2.2. Feature Selection Based on BAM

Let us consider a training dataset X and feature selection methods $\{FS_{1},\ldots ,FS_{t}\}.$ Let $X_{1},X_{2},\ldots ,X_{m}$ be bootstrapped samples from X, where $m\in Z_{>0}.$ Then, we apply $FS_{q},\;q=1,\ldots ,t$ on each $X_{s}$, s=1,⋯,m, which in turn generates feature scores ${\left\{\ell_{s1},\ldots ,\ell_{sp}\right\}}^{\left(q\right)}$ that correspond to the set of features $\{V_{1},\ldots ,V_{p}\}.$ Therefore, a score matrix $L^{\left(q\right)}=\left[\ell_{sj}^{\left(q\right)}\right]\in R^{m\times p}$ is generated after applying the feature selection method $FS_{q}$ on each bootstrap sample. In $L^{\left(q\right)}$, column *j* represents the $FS_{q}$ scores for variable $V_{j}$ over the *m* bootstrap sample datasets. Then, each column in the score matrix $L^{\left(q\right)}$ is normalized using min–max normalization; that is,

${\vec{L}}^{\left(q\right)}=\left[{\vec{\ell }}_{sj}^{\left(q\right)}\right]$, where ${\vec{\ell }}_{sj}^{\left(q\right)}=\frac{\ell_{sj}^{\left(q\right)}-\min_{s}\ell_{sj}^{\left(q\right)}}{\max_{s}\ell_{sj}^{\left(q\right)}-\min_{s}\ell_{sj}^{\left(q\right)}}$

**Figure 2:**
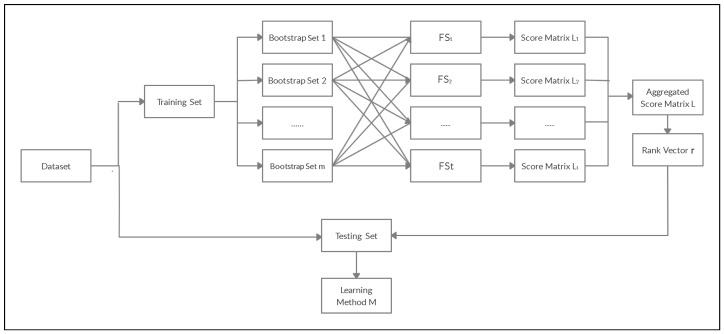
Framework for the Between Aggregation Method (BAM).

After the normalization, we use the arithmetic mean to combine the normalized aggregated scores across $FS_{q},\;q=1,\ldots t$ into one score matrix $\bar{\vec{L}}=\frac{\sum_{q=1}^{t}{\vec{L}}^{\left(q\right)}}{t}$. That is, column *j* in the score matrix $\bar{\vec{L}}$ represents the average column *j* between all considered feature selection methods $FS_{1},\ldots ,FS_{t}.$ Then, the final aggregated scores, $\{\bar{{\vec{\ell }}_{.1}},\ldots ,\bar{{\vec{\ell }}_{.p}\}},$ for the feature set $\left\{V_{1},\ldots ,V_{p}\right\}$ are the arithmetic mean of columns 1,…,p in $\bar{\vec{L}}.$ The rank vector $r=\left(r_{1},\ldots ,r_{p}\right),\;r_{j}\in \left\{1,2,\ldots ,p\right\}$ is assigned to the feature set $\left\{V_{1},\ldots ,V_{p}\right\}$ based on the final aggregated scores. The feature set is then sorted from the most to the least important based on the rank vector r. The top 100k% features are thus retained for further analysis. The flowchart in Figure 2 explains the BAM. The following gives the associated computational Algorithm 2.
**Algorithm 2BAM Algorithm:**Given a training dataset X with *p* features, a testing dataset T, feature selection methods $\{FS_{1},\ldots ,FS_{t}\},$ a threshold parameter k, and a learning algorithm M.
(i)For i=1,…,m, generate bootstrap samples, $X_{1},\ldots ,X_{m}$ of the training dataset X.(ii)For each feature selection method $FS_{q}\in \left\{FS_{1},\ldots ,FS_{t}\right\},$ get features score matrix $L^{\left(q\right)}.$(iii)Normalize the score matrices in (ii) as ${\vec{L}}^{\left(q\right)},\;q=1,\ldots ,t.$(iv)Use the arithmetic mean scores to combine the matrices in (iii) into one score matrix $\bar{\vec{L}}.$(v)Use the score matrix in (iv) to compute the aggregated scores $\{\bar{{\vec{\ell }}_{.1}},\ldots ,\bar{{\vec{\ell }}_{.p}\}}.$(vi)Based on the aggregated scores in (v), compute the corresponding rank vector $r=(r_{1},\ldots ,r_{p}).$(vii)Based on the rank vector r, keep the top 100k% of the variable set $\{V_{1},\ldots ,V_{p}\}.$(viii)Based on the selected feature set in (vii), use the testing dataset T and a cross-validation technique to train and test the model M.

## 3. Stability Analysis

In order to measure the stability of feature rankings for each feature selection method in $\left\{FS_{1},\ldots ,FS_{t}\right\}$, we implement the similarity-based approach proposed in [18]. This approach depends on the representation language of the produced feature rankings. Considering a training dataset X and a feature selection method FS, let $X_{1},X_{2},\ldots ,X_{m}$ be bootstrapped samples from X. Then, we apply FS on each $X_{s}$, s=1,⋯,m. This, in turn, produces any of the following three representations with respect to the feature set $\left\{V_{1},\ldots ,V_{p}\right\}$ and the sample dataset $X_{s}$:

- An importance scores vector $\ell_{s}=\left\{\ell_{s1},\ldots ,\ell_{sp}\right\},\ell_{sj}\in R$.
- A rank vector $r_{s}=\left\{r_{s1},\ldots ,r_{sp}\right\},\;r_{sj}\in \left\{1,2,\ldots ,p\right\}$.
- A subset of features represented by an index vector $w_{s}=\left\{w_{s1},\ldots ,w_{sp}\right\},\;w_{sj}\in \left\{1,0\right\}$, where 1 indicates feature presence and 0 indicates feature absence.

Naturally, it is possible to transform any feature importance scores vector *ℓ* into a rank vector r by sorting the importance scores. On the other hand, a rank vector r may be converted into an index vector w by selecting the top 100k% features. The most common way to quantify the stability of a feature selection method is by simply taking the average of similarity comparisons between every pair of feature rankings derived from the different bootstrap samples as follows:

(1) $$Stability=\frac{2}{m(m-1)}\sum_{s=1}^{m-1}\sum_{v=s+1}^{m}\Phi \left(f_{s},f_{v}\right)$$

where $\Phi (f_{s},f_{v})$ is the similarity measure between a pair of feature rankings from any two training samples $X_{s},X_{v}$ (1⩽s,v⩽m). Note that the feature rankings $\left(f_{s},f_{v}\right)$ can be represented as a pair of importance scores vectors, rank vectors or index vectors. Moreover, the multiple $\frac{2}{m(m-1)}$ stems from the fact that there are $\frac{m(m-1)}{2}$ possible pairs of feature rankings between the total *m* samples.

Several similarity measures have been introduced in the literature [8]. In this paper, we use some popular measures of similarity for each of the representations described above. Accordingly, we will use feature selection to produce importance score vectors $\left\{\ell_{1},\ldots \ell_{m}\right\}$ that will be converted into rank vectors $\left\{r_{1},\ldots r_{m}\right\}$ and index vectors $\left\{w_{1},\ldots w_{m}\right\}$. We implement the following:

i. Pearson’s correlation coefficient: In the case of similarity between two importance score vectors $\left(\ell_{s},\ell_{v}\right)$ produced by one of the feature selection methods, the Pearson’s correlation coefficient computes the similarity measure as
  (2) $$\Phi_{PCC}\left(\ell_{s},\ell_{v}\right)=\frac{\sum_{j=1}^{p}\left(\ell_{sj}-\mu_{s}\right)\left(\ell_{vj}-\mu_{v}\right)}{\sqrt{\sum_{j=1}^{p}{\left(\ell_{sj}-\mu_{s}\right)}^{2}{\left(\ell_{vj}-\mu_{v}\right)}^{2}}},$$
  where $\ell_{s}$ is the row *s* in the score matrix L; that is, the feature importance scores that correspond to the set of features $\left\{V_{1},\ldots ,V_{p}\right\}$, obtained from $X_{s}$. Furthermore, $\mu_{s}$ is the mean of the row vector $\ell_{s}$. Here, $\Phi_{PCC}\left(\ell_{s},\ell_{v}\right)\in \left[-1,1\right].$
ii. Spearman’s rank correlation coefficient: With regard to the similarity between two rank vectors $\left(r_{s},r_{v}\right)$ produced by one of the feature selection methods, Spearman’s rank correlation coefficient measures the similarity between the two rank vectors as
  (3) $$\Phi_{SRCC}\left(r_{s},r_{v}\right)=1-\frac{6\sum_{j=1}^{p}{\left(r_{sj}-r_{vj}\right)}^{2}}{p(p^{2}-1)},$$
  where $r_{s}$ is the rank vector that corresponds to the set of features $\left\{V_{1},\ldots ,V_{p}\right\}$, such that $r_{s}$ is derived from $\ell_{s}$. Here, $\Phi_{SRCC}(r_{s},r_{v})\in \left[-1,1\right].$
iii. Canberra’s distance: Another measure used to quantify the similarity between two rank vectors $\left(r_{s},r_{v}\right)$ is Canberra’s distance [19]. This metric represents the absolute difference between two rank vectors as
  (4) $$\Phi_{CD}\left(r_{s},r_{v}\right)=\sum_{j=1}^{p}\frac{|r_{sj}-r_{vj}|}{|r_{sj}\left|+\right|r_{vj}|}.$$
  For easier interpretation, Canberra’s distance is normalized by dividing by *p*.
iv. Jaccard’s index: Jaccard’s index measures the similarity between two finite sets; it is taken as the size of the intersection divided by the size of the union of the two sets. Given the index vectors $\left(w_{s},w_{v}\right)$ used to represent the two sets, Jaccard’s index is given by
  (5) $$\Phi_{JI}\left(w_{s},w_{v}\right)=\frac{|w_{s}\cap w_{v}|}{|w_{s}\cup w_{v}|}=\frac{|w_{s}\cap w_{v}|}{|w_{s}\left|+\right|w_{v}\left|-\right|w_{s}\cap w_{v}|},$$
  $\Phi_{JI}\left(w_{s},w_{v}\right)\in \left[0,1\right].$

In addition to the stability scores based on similarity, one can compute the average standard deviation of the feature importance scores across all bootstrap samples for every feature selection method. By definition, the standard deviation measures the dispersion or instability of the feature selection scores under different training bootstraps. Similar to the work in [20], we define the average standard deviation given a feature selection method FS as

(6) $$ASD=\frac{1}{p}\sum_{j=1}^{p}SD\left(c_{j}\right),$$

where $c_{j}$ represents column j in the standardized score matrix $\vec{L}$. In other words, $SD(c_{j})$ is the standard deviation of the standardized FS importance scores for variable $V_{j}$ over the *m* bootstrap samples. Generally, a low average standard deviation would imply high stability, whereas a high average standard deviation would suggest lower stability.

## 4. Experimental Evaluation

### 4.1. Experimental Datasets

Without the loss of generality of the applicability of the proposed framework, the experimental evaluation in this study focuses on the binary classification conferred in the datasets illustrated in Table 1. The target variable for each dataset encompasses two classes (e.g., “males” and “females”). Here, the class refers to the group categorization of some observations under the nominal target variable Y (i.e., the value of Y). The datasets contain both numerical and nominal features with various dimensions and numbers of observations. Overall, the number of features across datasets ranges from 34 to 309, while the number of observations ranges from 351 to 6598 observations. The features/observations ratio ranges from as low as 0.007 to as high as 0.194. Furthermore, the binary class distribution spans from deeply imbalanced to perfectly balanced. On that account, these datasets provide an interesting benchmark for investigating the performance of the proposed framework and its characteristics.

### 4.2. Experimental Design

In this experiment, we compared the performance of the two aggregation methods: WAM and BAM. The experimental environment was Windows 10, 64-bit, 16 GB RAM, Intel(R) Xeon E-2124 (3.30 GHz). For the implementation of the proposed framework (Section 2), we selected four filter selection methods: Information Gain (IG), Symmetrical Uncertainty (SU), Minimum Redundancy Maximum Relevance (MRMR) and the Chi-squared method (CS). For the Chi-squared method, numeric features were discretized based on a fixed-width binning. Each dataset was divided into training and testing datasets, where two-thirds of the dataset was used to obtain the feature rankings and one third was used for testing. For the training phase, m=1000 bootstrap samples were used. These bootstraps were utilized to obtain final rank vectors using the aggregation of feature importance scores within each feature selection method (WAM algorithm) and between the different feature selection methods (BAM algorithm). For the testing stage, 10 different *k* thresholds were used, resulting in subsets containing the top {10%,20%,⋯,100%} of the total features. Here, k=100% refers to the baseline model where all features were used and none of the feature selection methods were implemented. We should note here that other values of *k* can be chosen. The existing literature in this regard provides some guidelines on how to choose *k* for some specific scenarios. For example, we refer interested readers to [21,22,23,24,25]. However, it is difficult to generalize directives on the basis of specific cases, and in practice, several values of *k* were chosen and the classification accuracy using cross-validations or independent test data was used to evaluate the quality of the chosen subsets [26,27].

In the experiment, a five-fold cross-validation procedure was implemented in the testing phase. Accordingly, we divided the testing set into five stratified samples. Thus, the five folds were selected such that the distribution of the target variable Y was approximately equal in each of the folds. For instance, given a testing data with 500 observations such that the distribution of the binary target variable was 7:3, each fold in the stratified five-fold cross-validation would be expected to have 70 observations in one class and 30 in the other. Accordingly, stratification was used to ensure that each class was equally represented among the different folds. For every iteration, one of these five stratified samples was used as a testing set, while the remaining four samples were used to train the model. On each iteration, the testing sample was used to evaluate the performance of the selected feature subsets based on the following classification algorithms:

- Logistic regression: A statistical model used to model the probability of the occurrence of a class or an event using a logistic (sigmoid) function. It is a widely used classification algorithm in machine learning. The objective of logistic regression is to analyze the relationship between the categorical dependent variable and a set of independent variables (features) in order to predict the probability of the target variable. The maximum likelihood estimation method is usually used to estimate the logistic regression coefficients.
- Naive Bayes: A probabilistic classifier based on the Bayes theorem [28]. It assumes that the occurrence of each input feature is independent from other features. It can be used for both binary and multiclass classification problems. Due to its simplicity, it is a fast machine learning algorithm which can be used with large datasets.
- Random Forest: An ensemble model of decision trees in which every tree is trained on a random subsample to provide class prediction. The subsamples are drawn with replacements from the training dataset. The results from all the decision trees are then averaged to yield the model prediction [29]. Random Forest is useful to prevent over-fitting, but it can be complex to implement.
- Support Vector Machine (SVM): A supervised learning algorithm in which each observation is plotted as a point in p-dimensional space (with p being the number of features). SVM aims to identify the optimal hyperplane which segregates the data into separate classes. The selected hyperplanes thus maximize the distance between data points of different classes [30].

The entire experimental framework was performed using the open-source statistical programming language R. Note that features of near-zero variance were removed prior to the analysis. After the final feature subsets were selected and utilized in building each classifier across the five-folds, we evaluated the performance of the classifiers through the estimation of the area under the receiver operator curve (ROC) [31], hereafter referred to as the AUC. The AUC is a popular metric used to evaluate a model’s ability to distinguish between classes.

### 4.3. Discussion of the Results

**Figure A1:**
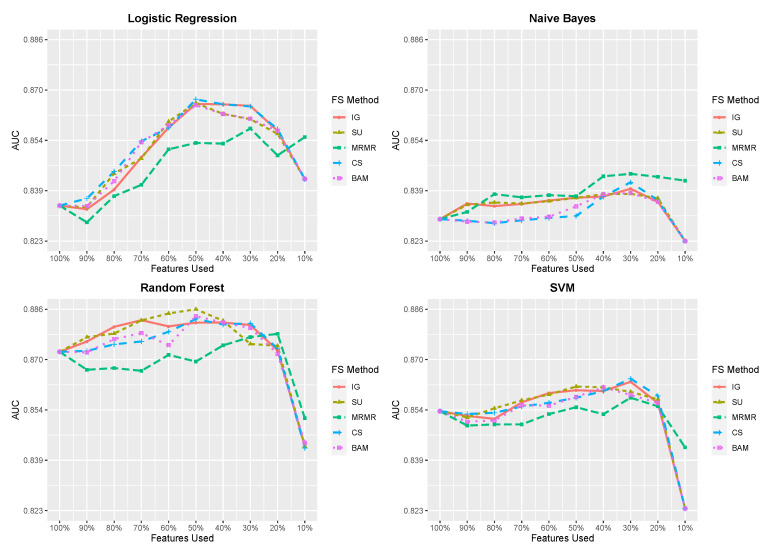
Jasmine dataset classification results. Running time = 1838 s (Within Aggregation Method (WAM)); 1840 s (Between Aggregation Method (BAM)).

**Figure A2:**
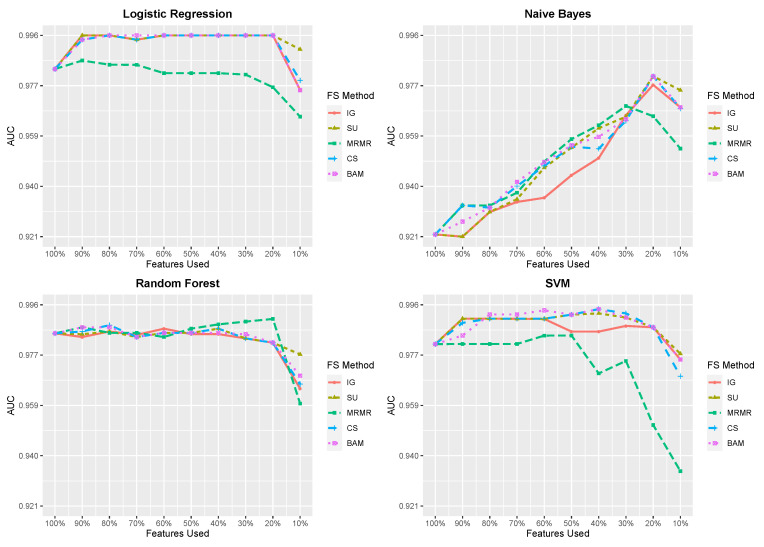
Spectrometer dataset classification results. Running time = 884 s (WAM); 889 s (BAM).

**Figure A3:**
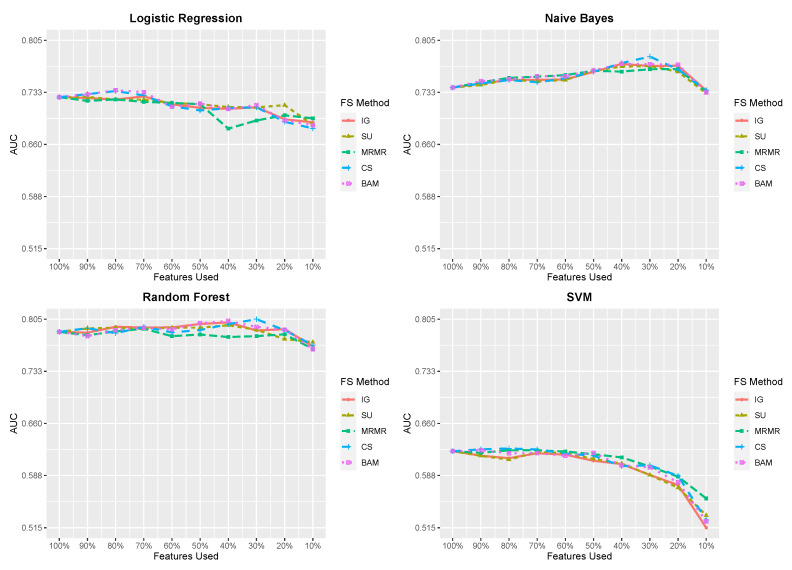
Image dataset classification results. Running time = 1816 s (WAM); 1843 s (BAM).

**Figure A4:**
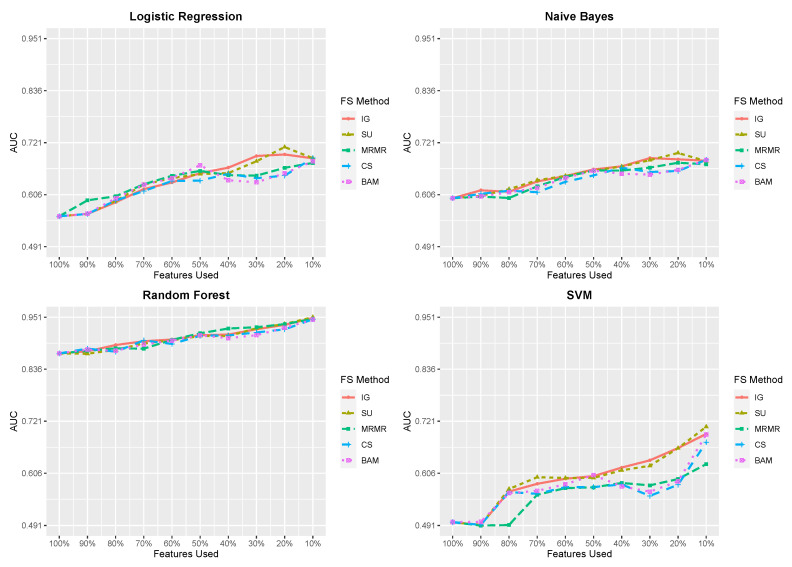
Fri classification results. Running time = 996 s (WAM); 1007 s (BAM).

**Figure A5:**
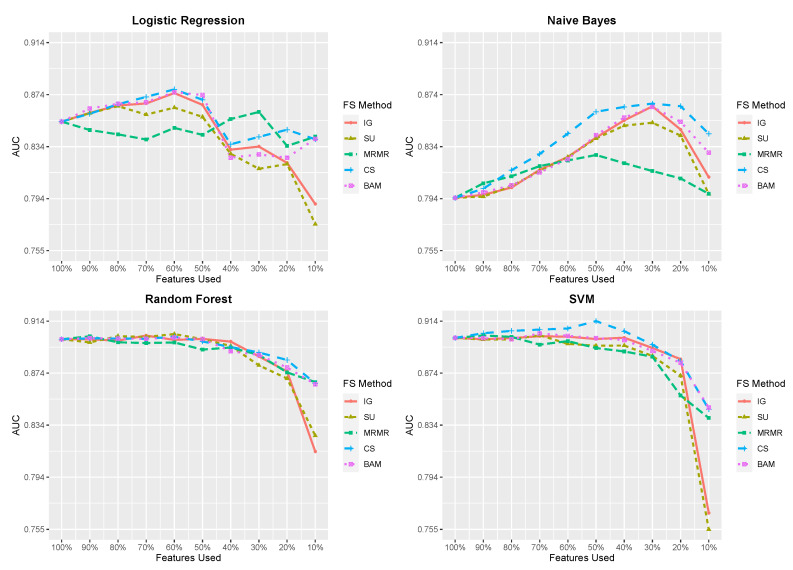
Scene dataset classification results. Running time = 4277 s (WAM); 4332 s (BAM).

**Figure A6:**
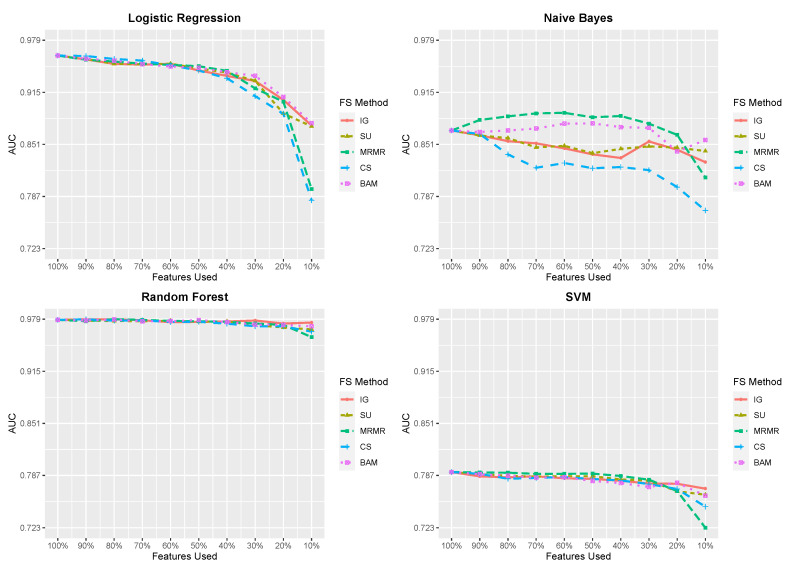
Musk dataset classification results. Running time = 4307 s (WAM); 4379 s (BAM).

**Figure A7:**
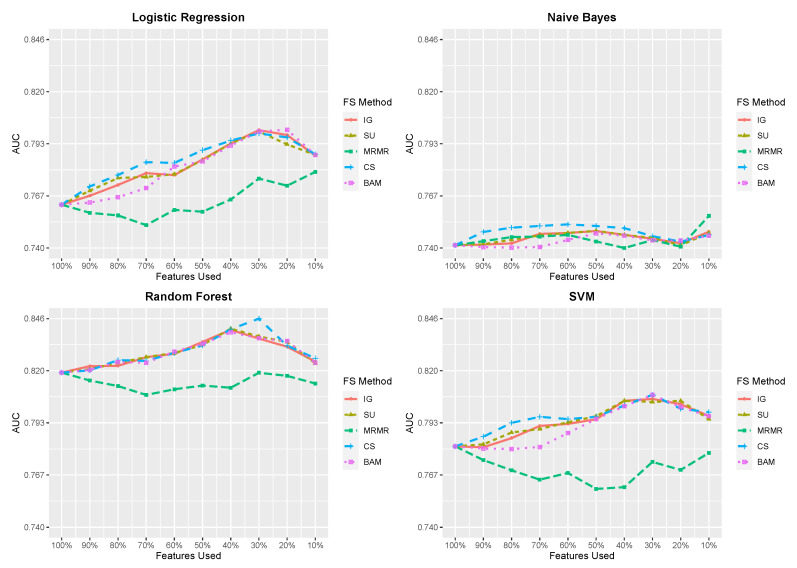
Philippine dataset classification results. Running time = 7595 s (WAM); 7601 s (BAM).

**Figure A8:**
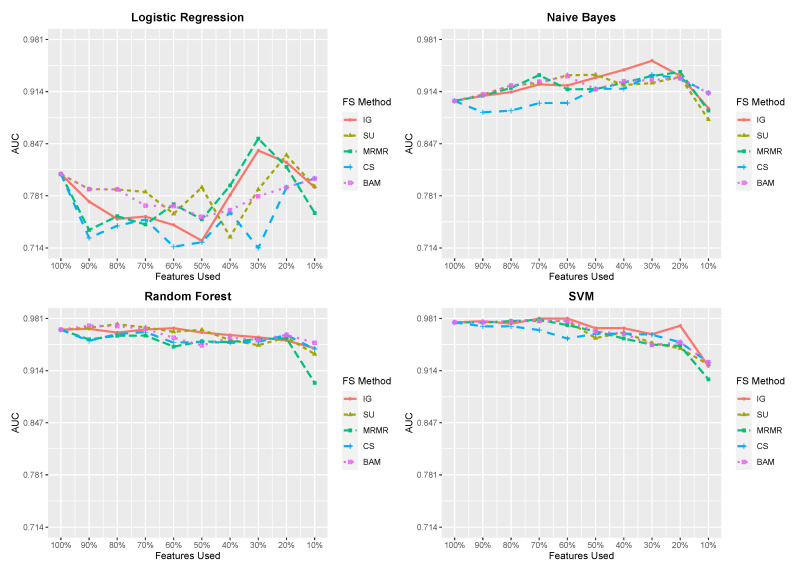
Ionosphere dataset classification results. Running time = 377 s (WAM); 377 s (BAM).

**Figure A9:**
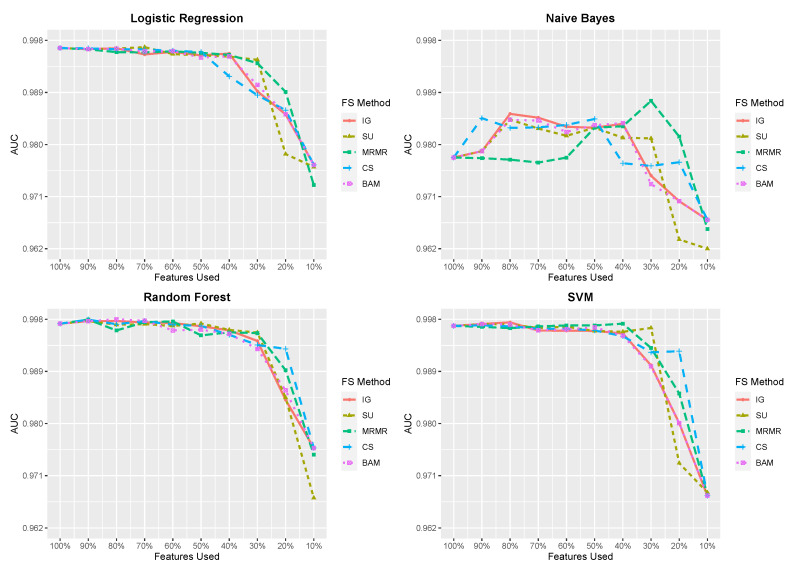
Optdigits dataset classification results. Running time = 1112 s (WAM); 1117 s (BAM).

**Figure A10:**
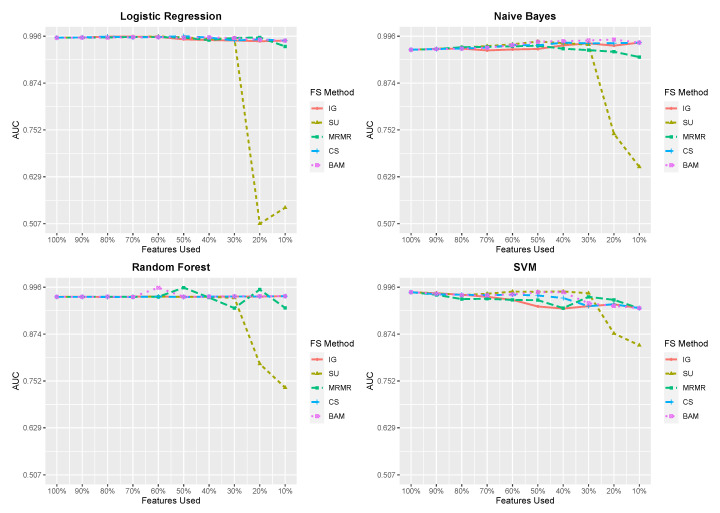
Satellite dataset classification results. Running time = 786 s (WAM); 799 s (BAM).

**Figure A11:**
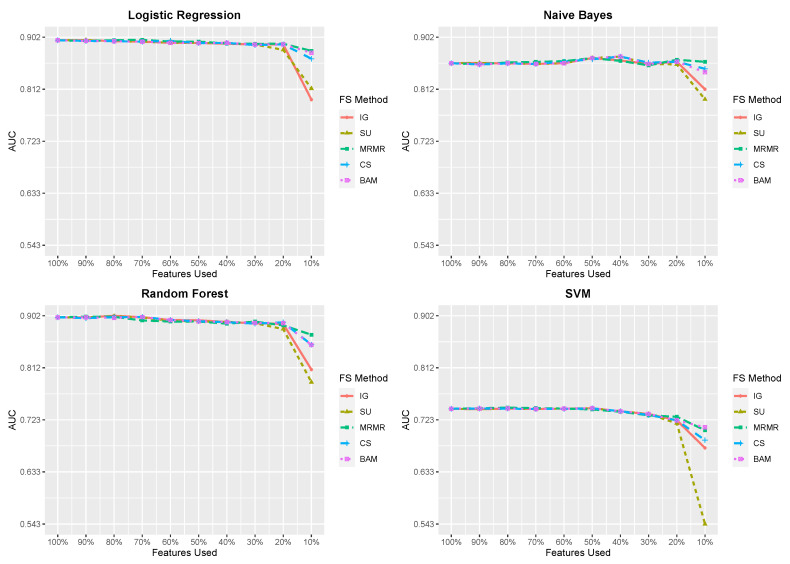
Ada dataset classification results. Running time = 698 s (WAM); 698 s (BAM).

**Figure A12:**
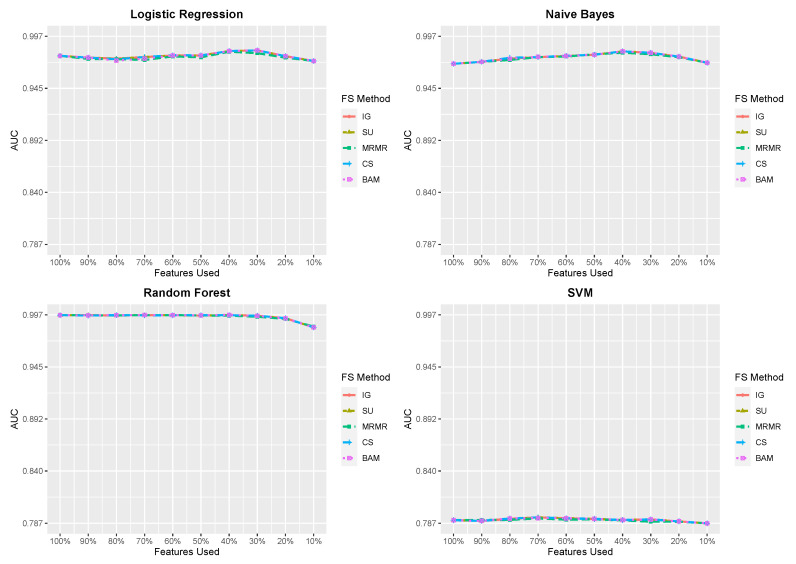
Splice dataset classification results. Running time = 1009 s (WAM); 1019 s (BAM).

**Figure A13:**
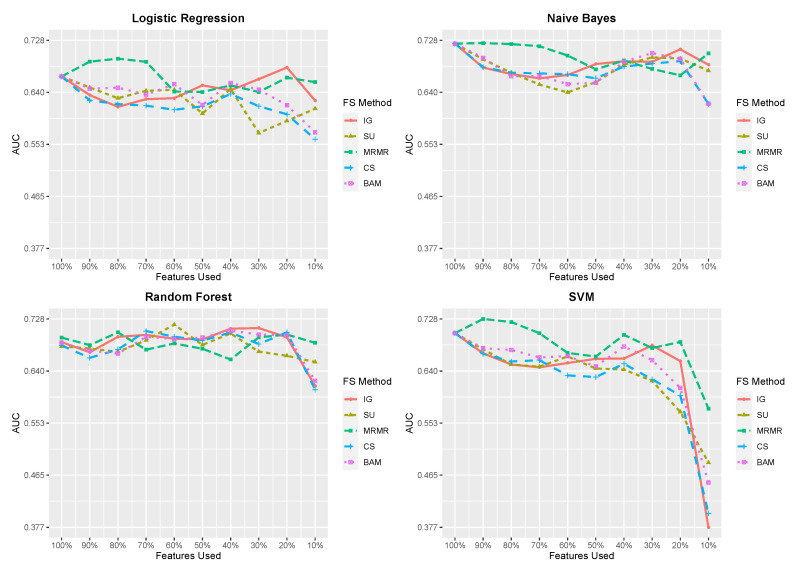
HIVA dataset classification results. Running time = 7622 s (WAM); 7624 s (BAM).

Figure A1, Figure A2, Figure A3, Figure A4, Figure A5, Figure A6, Figure A7, Figure A8, Figure A9, Figure A10, Figure A11, Figure A12 and Figure A13 in the Appendix A shows the resulting AUC values after applying the WAM and the BAM to each dataset. The results of WAM are depicted by the curves corresponding to the individual feature selection methods: IG, SU, MRMR and CS. On the other hand, the results of BAM are represented by the single BAM curve in each plot. The AUC values, averaged over the five-folds in the cross-validation, are plotted against the 10 different k100% thresholds (ranging between 10–100% of features used) in order to reveal the classification performance for different feature subsets in the testing stage. The running times for WAM and BAM, respectively, are also shown in Figure A1, Figure A2, Figure A3, Figure A4, Figure A5, Figure A6, Figure A7, Figure A8, Figure A9, Figure A10, Figure A11, Figure A12 and Figure A13. The average running time for WAM was 2563 s and that for BAM was 2579 s. Overall, BAM was marginally slower than WAM across all datasets because of the fact that BAM involves an additional aggregation step between the different feature selection methods. It should be emphasized that the computational costs of the proposed framework are mostly dependent on the feature selection methods used and the dataset composition. In the following two subsections, we analyze and compare the performance of the WAM and BAM algorithms in terms of their classification accuracy and their identification of optimal feature subsets (Section 4.3.1). In Section 4.3.2, we analyze and compare the stability behavior of the two algorithms. Furthermore, the association between stability and accuracy is discussed.

#### 4.3.1. Classification Performance

In most datasets, we note that the ensembling of bootstrap samples and aggregation of feature importance scores within and between feature selection methods improved the baseline (k=100% features are used) classification performance. In particular, the effectiveness of WAM was noticeable under the Naive Bayes classifier, where the AUC for most aggregated feature selection methods showed a steeper increase than in other classifiers across the six datasets. For instance, the Naive Bayes accuracy increased from around 0.79 baseline AUC to nearly 0.86 in the Scene dataset (Figure A5) and from the baseline of 0.60 AUC to nearly 0.70 in the Fri dataset (Figure A4). Following the removal of the least significant features, a similar increase in the performance of the logistic regression model could be observed in the Jasmine and Philippine datasets (Figure A1 and Figure A7). At baseline performance, it seems that the Random Forest algorithm performed better than the other classifiers, with SVM achieving the lowest AUC scores in the majority of cases. Although the aggregated feature selection methods present some highly changing patterns depending on the number of features retained, the general trend showed an increase in accuracy scores. Moreover, the variability in the AUC scores between the different feature selection methods tended to be more pronounced in the high-dimensional datasets (e.g., Philippine, Jasmine, Scene, HIVA) in comparison to the low-dimensional datasets (e.g., Satellite, Ada, Splice).

Likewise, it can be observed that BAM also improved the baseline accuracy of the classification models, especially under logistic regression. For instance, BAM improved the logistic regression AUC scores from around 0.83 at baseline to over 0.86 in Jasmine (Figure A1) and from around 0.55 at baseline to nearly 0.67 in Fri (Figure A4).

A general observation from the figures is that the aggregated feature selection methods under the WAM outperformed the BAM. In particular, for most of the datasets, there was at least one feature selection method aggregated under WAM that produced better AUC values than BAM. For instance, it is clear that the aggregated Chi-squared (CS) method outperformed BAM in the Philippine and Scene datasets (Figure A7 and Figure A5, respectively). In other datasets such as Fri (Figure A4) or Ionosphere (Figure A8), the aggregated IG and SU tended to produce the highest AUC scores across the classifiers. The performance of BAM appeared to be comparable to that of the aggregated feature selection methods in the datasets Image, Satellite, and Spectrometer. BAM was among the best-performing methods with respect to AUC in these datasets. The figures show that, in general, the aggregated IG and CS demonstrate the greatest correlation with BAM curves.

For the selection of the optimal k100% threshold based on AUC values, the WAM and BAM produced nearly consistent results. In the Philippine, Jasmine and Musk datasets, there was a noticeable pattern in which the classification AUC dropped sharply around the 30% mark, indicating that removing more than 70% of the features results in the reduced accuracy of the trained model. Nevertheless, the exact feature reduction percentage is dependent on the dataset used. In the Scene dataset, most of the classifiers agreed on retaining around 50% of the top features. In the Splice and Optidigits datasets, the optimal k100% was 70%, while in the Spectrometer and HIVA datasets, this was almost 80% of the features.

It should be mentioned that the experimental results in this paper affirmed the fact that the performance of a given feature selection method is data-dependent. Overall, none of the feature selection methods produced the best AUC values across all datasets. For example, the aggregated MRMR showed the worst performance across multiple classifiers in the Scene, Spectrometer and Philippine datasets, whereas in the Optdigits dataset (Figure A9), it showed improved performance when most features were removed. A similar pattern was seen for the aggregated Chi-squared feature selection method, which showed the best performance in a number of datasets (e.g., Image, Scene, Philippine) and worse performance in others (e.g., Fri, Ionosphere). The BAM curve was seen to be the middle-best performing technique. This is of course because the BAM scores are simply the aggregated averages over the individual feature selection methods. In most cases, however, there was an obvious overlap in the AUC scores between the different feature selection techniques resulting from the fact that dominant features were able to maintain similar rankings under different feature selection algorithms.

In summary, the analysis of the AUC results reveals that WAM can be used as a powerful tool to help practitioners to identify the most suitable feature selection method for a given data set. It can also guide the selection of the optimal level of feature reduction while achieving the maximum level of learning accuracy.

#### 4.3.2. Stability Analysis Results

Table 2 presents the results of the stability analysis of the four aggregated filters using WAM and BAM. Stability scores derived from the pairwise Pearson’s correlation coefficient are calculated using the importance scores in the matrix $L=\left[\ell_{sj}\right]\in R^{m\times p}$ resulting from applying the feature selection method FS on every bootstrap sample. The calculated Pearson’s correlation coefficients are then averaged over all possible pairs. On the other hand, the averaged pairwise Spearman’s rank correlation coefficients and Canberra’s distance are calculated from the rank matrix $R=\left[r_{sj}\right]\in R^{m\times p}$, which is obtained by sorting the features from most to least important based on their importance scores $L=\left[\ell_{sj}\right]\in R^{m\times p}$. The Jaccard’s index is computed from the top 25% ranked feature subsets (represented by index vectors). Finally, the average standard deviation (ASD) is computed using the importance scores, after normalization, averaged over the 1000 bootstraps.

The bolded scores in Table 2 represent the best stability value for each dataset. In the Jaccard’s index, Spearman’s, and Pearson’s correlation coefficient, this refers to the highest stability score, whereas for Canberra’s distance and ASD, it represents the lowest stability score. Unsurprisingly, none of the feature selection methods demonstrated the best stability behavior consistently across the 13 datasets. In other words, the stability of the aggregated feature selection methods was data-dependent. As such, even though all the stability measures agreed on a “winning” feature selection method in some datasets, it is not surprising that these methods varied throughout. For example, IG was the most stable method across all stability measures in the Ada and Philippine datasets, but Symmetrical Uncertainty was the most stable in Ionosphere and MRMR in the Spectrometer and HIVA datasets. Thus, none of the aggregated feature selection methods can be declared the most stable in every measure.

Contrasting the stability behavior of the BAM to that of the WAM, Table 2 shows that at least one feature selection method of the four feature selection methods used generally exhibited higher stability when aggregated using WAM than when aggregated using BAM. With the exception of the Image dataset, the BAM stability scores were seen to be the middle-most stable across every experimental stability measure. For the Image dataset, the BAM stability outperformed the stability of all the aggregated feature selection methods. It is noteworthy that BAM also achieved the best classification accuracy in the Image dataset (see Figure A3). Similarly, the IG and Chi-squared methods, which demonstrated the highest stability in the Fri and Scene datasets, also achieved the best classification performance in these two datasets, respectively (see Figure A4 and Figure A5). A similar pattern can be seen with respect to the higher stability of MRMR in the Optdigits and HIVA datasets. These observations suggest that there may exist a positive association between the classification performance of a feature selection method and its stability behavior. Intuitively, this presumed association depends on the stability metrics used and also on the characteristics of the dataset and the learning algorithm—a topic that constitutes an interesting line of further research.

In summary, although BAM demonstrates a comparable stability behavior to that for each single feature selection method under WAM, it appears that, in 11 out of 12 datasets used in this paper, the stability of at least one individual method outperformed BAM. Interestingly, there appears to be a positive association between the classification performance of a feature selection method and its stability behavior. A feature selection method that outperforms others in terms of classification accuracy may also outperform them in terms of stability behavior.

## 5. Conclusions

Over the years, datasets have grown increasingly large in terms of their size and dimensionality. As a result, feature selection has become a necessary reprocessing tool in machine learning applications and the focus of a wide range of literature and research across many domains. This study has explored the full potential of a bootstrap ensemble approach for feature selection in which the ensemble aggregation is performed within and between the multiple feature selection methods.

The extensive experimental analysis of 13 different datasets selected from different domains has demonstrated that the Within Aggregation Method (WAM) is highly efficient in guiding the selection of the most suitable feature selection method for a given problem. As for reducing the dimensionality of the problem, our analysis showed that WAM and the Between Aggregation Method (BAM) were comparable in determining the optimal percentage reduction in the number of features. They are also comparable in terms of the computational costs. It important to emphasize that optimizing the feature subsets and the computational costs of the techniques depends largely on the dataset characteristics and learning algorithm implemented.

In terms of stability, the WAM demonstrated better stability behavior than BAM in most datasets (11 out of 12). Overall, the BAM stability scores fell in the middle range of the computed values on each of the score-based and rank-based stability metrics, suggesting a desirable stability behavior of the method.

The experimental analysis also showed that there exists a positive association between the classification performance of a feature selection method and its stability behavior. In other words, the feature selection method that outperforms others in terms of classification accuracy may also outperform them in terms of stability behavior. This association can, however, depend on the stability metric used, the learning algorithm and the characteristics of the dataset. Due to the extent to which the feature selection results in terms of learning accuracy and stability can depend on the data composition and learning algorithm, it is essential that both BAM and WAM methods be implemented in order to achieve a better understanding of and more useful insights into the underlying application domain.

The observed association between learning accuracy and stability indeed merits further investigation in the future. Additionally, in future work, we will extend the application of the framework to other types of feature selection methods, such as wrappers. Particular interest will be given to the application using a mix of filter and wrapper feature selection methods and establish a wider scope of comparisons to better evaluate the ensemble framework. Furthermore, datasets with diverse performance and various learning algorithms will be implemented. Another line of future research would be the application and analysis of additional aggregation methods, such as the median or geometric mean, and the comparison of them against the mean aggregation used in this paper.

## Figures and Tables

**Table 1:** Description of datasets.

Dataset Name and Source	No. Observations	No. Features	No. Classes	Dimensionality *
Jasmine ${}^{1}$	2984 (1492/1492)	145	2	0.048592
Spectrometer ${}^{2}$	531 (476/55)	103	2	0.193974
Image ${}^{2}$	2000 (1420/580)	140	2	0.07
Fri ${}^{2}$	1000 (564/436)	101	2	0.101
Scene ${}^{3}$	2407 (1976/431)	295	2	0.122559
Musk ${}^{4}$	6598 (5581/1017)	170	2	0.025765
Philippine ${}^{1}$	5832 (2916/2916)	309	2	0.052984
Ionosphere ${}^{4}$	351 (126/225)	34	2	0.096866
Optdigits ${}^{2}$	5620 (572/5048)	64	2	0.011388
Satellite ${}^{2}$	5100 (75/5025)	37	2	0.007255
Ada ${}^{1}$	4147 (1029/3118)	49	2	0.011816
Splice ${}^{2}$	3190 (1535/1655)	62	2	0.019436
HIVA ${}^{2}$	4229 (149/4080)	1617	2	0.382359

* Dimensionality is the ratio of features to number of observations. Superscripts indicate the data sources as follows: ^1^ automl.chalearn.org, ^2^ www.openml.org, ^3^ mulan.sourceforge.net, ^4^ archive.ics.uci.edu.

**Table 2:** Stability analysis results across all datasets. MRMR: Minimum Redundancy Maximum Relevance.

Dataset	Stability Measure	Information Gain	Symmetrical Uncertainty	MRMR	Chi-Squared	BAM
Jasmine	Average Pearson Correlation	0.299705	0.258270	**0.902748**	0.360289	0.335806
	Average Spearman Rank Correlation	0.319009	0.378017	**0.730414**	0.231101	0.334655
	Average Jaccard’s Index	0.254683	**0.319411**	0.308712	0.286586	0.252964
	Average Canberra Distance	0.278113	0.269500	**0.124180**	0.337122	0.294955
	Average Standard Deviation	0.744817	0.753504	**0.149429**	0.747054	0.765530
Spectrometer	Average Pearson Correlation	0.898348	**0.942554**	0.698045	0.916711	0.927582
	Average Spearman Rank Correlation	0.831602	**0.837903**	0.765404	0.818745	0.833578
	Average Jaccard’s Index	0.759908	**0.917055**	0.436905	0.851790	0.872271
	Average Canberra Distance	0.153674	**0.152607**	0.171177	0.186988	0.182109
	Average Standard Deviation	0.308687	**0.223475**	0.456242	0.281311	0.258388
Image	Average Pearson Correlation	0.768404	0.760257	0.461867	0.784970	**0.793634**
	Average Spearman Rank Correlation	0.702970	0.690212	0.541442	**0.716971**	0.671209
	Average Jaccard’s Index	0.534739	0.514374	0.275411	0.557456	**0.560470**
	Average Canberra Distance	**0.140367**	0.142500	0.236440	0.242640	0.254949
	Average Standard Deviation	0.437907	0.462153	0.643365	0.459268	**0.434591**
Fri	Average Pearson Correlation	**0.969642**	0.912108	0.955966	0.818795	0.948385
	Average Spearman Rank Correlation	0.458619	**0.459933**	0.301028	0.327351	0.308725
	Average Jaccard’s Index	**0.600791**	0.600791	0.263723	0.288660	0.280655
	Average Canberra Distance	0.057812	**0.057752**	0.329030	0.328557	0.330035
	Average Standard Deviation	**0.146339**	0.268018	0.201306	0.422568	0.218823
Scene	Average Pearson Correlation	0.898425	0.887953	0.652895	**0.933014**	0.908673
	Average Spearman Rank Correlation	0.871633	0.863620	0.705409	**0.904599**	0.881263
	Average Jaccard’s Index	0.725580	0.718157	0.429004	**0.834032**	0.761022
	Average Canberra Distance	**0.150622**	0.156575	0.206240	0.169175	0.182344
	Average Standard Deviation	0.309285	0.328335	0.501868	**0.253048**	0.296812
Musk	Average Pearson Correlation	0.953028	0.939819	**0.983622**	0.972910	0.971086
	Average Spearman Rank Correlation	0.897172	0.920754	**0.978164**	0.958189	0.932817
	Average Jaccard’s Index	0.254683	**0.319411**	0.308712	0.286586	0.252964
	Average Canberra Distance	0.278113	0.269500	**0.124180**	0.337122	0.294955
	Average Standard Deviation	0.198588	0.230549	**0.106096**	0.153881	0.164122
Philippine	Average Pearson Correlation	**0.992381**	0.987185	0.949337	0.974312	0.990140
	Average Spearman Rank Correlation	**0.948322**	0.945942	0.876291	0.794429	0.826292
	Average Jaccard’s Index	**0.907578**	0.895855	0.599476	0.882073	0.898057
	Average Canberra Distance	**0.036655**	0.037883	0.123565	0.216403	0.199559
	Average Standard Deviation	**0.065557**	0.093865	0.194033	0.133756	0.090117
Ionosphere	Average Pearson Correlation	0.398351	0.583203	**0.803445**	0.689003	0.678480
	Average Spearman Rank Correlation	0.391580	0.583566	**0.779300**	0.634363	0.621247
	Average Jaccard’s Index	0.322984	0.418490	**0.588096**	0.511871	0.514258
	Average Canberra Distance	0.284348	0.254220	**0.185660**	0.245600	0.249635
	Average Standard Deviation	0.731482	0.606503	**0.397275**	0.546923	0.549206
Optdigits	Average Pearson Correlation	0.974733	0.956047	0.946192	**0.978112**	0.976264
	Average Spearman Rank Correlation	0.965357	0.959320	0.913443	**0.968890**	0.967125
	Average Jaccard’s Index	**0.776935**	0.687190	0.621800	0.699440	0.740367
	Average Canberra Distance	0.087271	0.094572	0.112731	**0.077847**	0.090535
	Average Standard Deviation	0.150498	0.196308	0.188786	**0.141531**	0.146916
Satellite	Average Pearson Correlation	0.962102	0.735536	0.735555	**0.962324**	0.932846
	Average Spearman Rank Correlation	0.913703	0.737037	0.886279	**0.941141**	0.912465
	Average Jaccard’s Index	**0.889171**	0.523733	0.579217	0.711644	0.540189
	Average Canberra Distance	0.128159	0.206656	**0.093391**	0.117366	0.126052
	Average Standard Deviation	0.189240	0.429693	0.289669	**0.186215**	0.235742
Ada	Average Pearson Correlation	**0.998732**	0.998655	0.997906	0.992348	0.998004
	Average Spearman Rank Correlation	**0.956392**	0.952797	0.823995	0.955461	0.952162
	Average Jaccard’s Index	**0.919947**	0.866222	0.607214	0.830739	0.863409
	Average Canberra Distance	**0.106299**	0.108989	0.155215	0.122942	0.125797
	Average Standard Deviation	**0.028835**	0.031522	0.029137	0.083938	0.042076
Splice	Average Pearson Correlation	0.992299	**0.993156**	0.990926	0.974606	0.989386
	Average Spearman Rank Correlation	0.841882	0.842385	0.738889	**0.843115**	0.836391
	Average Jaccard’s Index	0.761442	**0.762814**	0.597770	0.760529	0.742453
	Average Canberra Distance	0.187747	0.187556	0.224846	**0.187334**	0.190992
	Average Standard Deviation	0.070557	**0.065447**	0.081051	0.157357	0.096031
HIVA	Average Pearson Correlation	0.738293	0.764771	**0.866538**	0.746545	0.746723
	Average Spearman Rank Correlation	0.603392	0.621829	**0.804467**	0.648684	0.639793
	Average Jaccard’s Index	0.654280	0.583914	**0.752230**	0.618569	0.623986
	Average Canberra Distance	0.277170	0.2369058	**0.147782**	0.260381	0.252760
	Average Standard Deviation	0.457420	0.395126	**0.318563**	0.466903	0.456952
